# Gateway-Compatible CRISPR-Cas9 Vectors and a Rapid Detection by High-Resolution Melting Curve Analysis

**DOI:** 10.3389/fpls.2017.01171

**Published:** 2017-07-05

**Authors:** Cynthia J. Denbow, Samantha Lapins, Nick Dietz, Raelynn Scherer, Zachary L. Nimchuk, Sakiko Okumoto

**Affiliations:** ^1^Department of Plant Pathology, Physiology and Weed ScienceBlacksburg, VA, United States; ^2^Department of Biology, University of North CarolinaChapel Hill, NC, United States; ^3^Department of Soil and Crop Science, Texas A&M UniversityCollege Station, TX, United States

**Keywords:** CRISPR/Cas9, high-resolution melting curve analysis, gene editing, indel detection, genome editing

## Abstract

CRISPR-Cas9 system rapidly became an indispensable tool in plant biology to perform targeted mutagenesis. A CRISPR-Cas9-mediated double strand break followed by non-homologous end joining (NHEJ) repair most frequently results in a single base pair deletion or insertions (indels), which is hard to detect using methods based on enzymes that detect heteroduplex DNA. In addition, somatic tissues of the T1 generation inevitably contain a mosaic population, in which the portion of cells carrying the mutation can be too small to be detected by the enzyme-based methods. Here we report an optimized experimental protocol for detecting Arabidopsis mutants carrying a CRISPR-Cas9 mediated mutation, using high-resolution melting (HRM) curve analysis. Single-base pair insertion or deletion (indel) can be easily detected using this method. We have also examined the detection limit for the template containing a one bp indel compared to the WT genome. Our results show that <5% of mutant DNA containing one bp indel can be detected using this method. The vector developed in this study can be used with a Gateway technology-compatible derivative of pCUT vectors, with which off-target mutations could not be detected even by a whole genome sequencing.

## Introduction

CRISPR-Cas9 technology made targeted mutagenesis readily possible for many organisms. Unlike its predecessors, such as zinc-finger nucleases and transcription activator-like effector nucleases (TALENs), the CRISPR-Cas9 system does not require a customized protein for each target sequence, offering a far more versatile and cost-effective system (Baltes and Voytas, 2015; Belhaj et al., 2015).

CRISPR-Cas9-mediated double strand breaks repaired by the non-homologous end joining (NHEJ) pathway mostly result in a small deletion or insertion (indel) at the target site, of which one bp indels are the most frequent (Ma et al., 2015; Pan et al., 2016; Ren et al., 2016). While these small indels effectively cause loss-of-function through a frame shift if they are in a protein coding region, they cannot be detected using a DNA-agarose gel due to the small size shift.

Currently, the most commonly used method for detecting indels is the enzymatic mismatch cleavage (EMC) method (Yeung et al., 2005; Vouillot et al., 2015). A typical protocol would involve; (1) PCR-amplification of the target sequence, (2) Melting and hybridizing the resulting PCR fragment to create mismatched double-stranded DNA, and (3) Cleavage by the enzymes that specifically digest mismatched fragments, followed by detection with a DNA-agarose gel. This type of method is particularly effective for a relatively large indel; detection limit of 0.5–5% of the total population has been reported (Zhu et al., 2014; Vouillot et al., 2015). The enzyme utilized for this method, such as T7 endonuclease (T7E1) and CEL nuclease, however, tends to produce background due to non-specific exonuclease activities (Huang et al., 2012). In addition, one bp indels are more difficult to detect with EMC methods. T7E1 nuclease, which detects small indels better than CEL nuclease, detects a kink in the DNA double strands caused by additional bases (Declais and Lilley, 2008). Although detection of one-bp deletion using T7E1 has been reported, detection efficiency decreases for smaller indels (Vouillot et al., 2015; Zischewski et al., 2017), likely due to the lower degree of DNA distortion caused by smaller indels (Gohlke et al., 1994).

An alternative method is the polyacrylamide gel electrophoresis (PAGE)-based method, which takes advantage of the change in DNA migration due to the bulge structure. PAGE-based methods do not require an enzymatic digestion process, and the sensitivity is comparable to the EMC method. On the other hand, the shift in migration becomes harder to detect for one bp indels (Zhu et al., 2014).

High-resolution melting (HRM) analysis detects the decreased melting temperature in heteroduplex DNA fragments compared to the homoduplex ones. The HRM method offers multiple advantages over the EMC and PAGE methods (Wittwer et al., 2003; Wittwer, 2009; Fauser et al., 2014; Simko, 2016). The method does not require any additional pipetting step after the PCR reaction, and is very rapid (<10 min for post-PCR analysis). The fluorescent dye can be added to any PCR mix of choice, which is beneficial especially for plant researchers who might have a particular requirement for the PCR enzyme used (e.g., a polymerase that is resistant to polyphenols). Most importantly in the context of CRISPR/Cas9 based genome editing, HRM very reliably detects one bp indels (Dufresne et al., 2006; Declais and Lilley, 2008).

Here, we describe the method used in our laboratory to detect CRISPR/Cas9 based gene editing in *Arabidopsis thaliana*. We have developed a Gateway-compatible vectors for constructing a tandem repeat of two single guide RNA (sgRNA) sequences, which takes advantage of a set of Cas9 expression vectors published recently (Peterson et al., 2016). Using LC Green Plus, a dye that detects heteroduplex DNA at a higher sensitivity compared to other dyes, such as SYBR green (Wittwer et al., 2003), we show that HRM provides a rapid screening method for T1 mutants, and that as little as ~5% of mutant DNA carrying a one bp indel can be detected reliably.

## Materials and methods

### Gene constructs

The tandem U6 promoter-sgRNA cassettes flanked by attL1 and attL2 sites were created through gene synthesis and recombined into a modified pDONRzeof1 vector (Lalonde et al., 2010) in which the endogenous BbsI and SapI sites were removed by site-directed mutagenesis (Kunkel, 1985). A 293 bp-long U6 promoter was used to drive sgRNA expression. The resulting vector was named pDONRzeof1m-U6T. The procedure used to create CRISPR/Cas9 expression vector targeting At1g68170 and At1g25270 is represented in Supplemental Figure 1. The target sequences, shown in Table S1, were identified using the web-based tool CRISPR-P (http://cbi.hzau.edu.cn/crispr/, Lei et al., 2014). Two independent fragments, each containing one sgRNA targeting At1g68170, one U6 promoter and one target sequence for At1g25270 were amplified by PCR using pDONRzeof1m-U6T as the template (the primers used are shown in Table S2, At1g68170/27250-1 and 2). The fragments were then cloned into pDONRzeof1m-U6T, which provided the U6 promoter for the first sgRNA and the sgRNA sequence without the target sequence for the second sgRNA. The resulting constructs were then tandemly fused by excising the first tandem construct with SalI and EcoRV and inserting into the XhoI and Ecl136II sites in the second construct. The resulting construct was recombined into pMTN3164, which carries the CAS9 protein tagged with the human influenza hemagglutinin (HA) tag and N7 nuclear localization signal (Cutler et al., 2000) using the Gateway cloning method.

### Plant growth conditions and transformation

Arabidopsis plants (ecotype Col-0) were grown on soil under a 16 h light/8 h dark cycle, 50% humidity, and 22°C. Arabidopsis transformation was performed by infecting Arabidopsis influorescences with Agrobacterium GV3101 using the floral dip method (Clough and Bent, 1998). T1 transformants were selected on half-strength Murashige and Skoog medium without sucrose containing 20 μg/ml hygromycin under the growth condition above. After 2 weeks, transformed seedlings developed true leaves and roots, whereas non-transformants turned white and died. Transformation efficiency was ~1/2,000–1/5,000, comparable to the efficiency observed by other groups (Clough and Bent, 1998; Ghedira et al., 2013).

### Genomic DNA extraction

Genomic DNA from Arabidopsis was isolated by a protocol described in Murray and Thompson (1980), with some modifications. A single Arabidopsis leaf was macerated in liquid N_2_, then incubated in a 1:1 mixture of chloroform/IAA and extraction buffer (2% CTAB, 100 mM Tris-HCl pH 8.0, 1.4 M NaCl) at 65°C for 30 min. The aqueous phase after the centrifugation step was extracted again with chloroform/IAA, and mixed with an equal volume of isopropanol to precipitate the genomic DNA. The pellets were dissolved in 50 μL of TE buffer containing RNase A at 0.1 mg/mL and incubated at 37°C for 30 min. The genomic DNA was dissolved in 400 μL of 1 M CsCl, precipitated by adding 800 μL of ethanol, then dissolved in 50 μL TE buffer.

### PCR using LC green plus dye

PCR was performed using Phire Hot Start II DNA Polymerase (Thermo Fisher Scientific, USA) according to the manufacturer's protocol with a few modifications; the reaction contained 1/10 vol of LC Green Plus dye (BioFire Defense, USA), and 20 μL mineral oil was added to each reaction to prevent condensation on the plate seal. A 1/10 dilution of the genomic DNA (ranging from 5 to 50 ng/μL) was used as a template for PCR. The primers used for the detection of gene editing activities are shown in Table S2 (pairs At1g68170-1 and 2, At1g25270-1 and 2). For testing varied ratios of mutant and WT DNA as the template, the genomic DNA from the mutant and WT were added at the total concentration of 0.5 ng/μL to the PCR reaction. The finished PCR products were analyzed by one of two melt curve approaches; either by HRM using the LightScanner system (BioFire Defense, USA) or the meltcurve function of the ABI7500 qPCR system (Applied Biosystems USA).

### Melt curve analysis

High-resolution melting (HRM) analysis was performed using LightScanner software (BioFire Defense, USA). The data was normalized by visually identifying the baseline regions below and above the melting temperature, which was used for the linear baseline correction method previously described (Palais and Wittwer, 2009). For the data obtained using the ABI7500 qPCR system, the data were normalized using the method described in Palais and Wittwer (2009), followed by Savitzky-Golay algorithm using the filter width of 2n +1 = 3 and a quadratic polynomial fit (Savitzky and Golay, 1964).

## Results and discussion

### Assembly U6Promoter-sgRNA repeats

**Figure 1:**
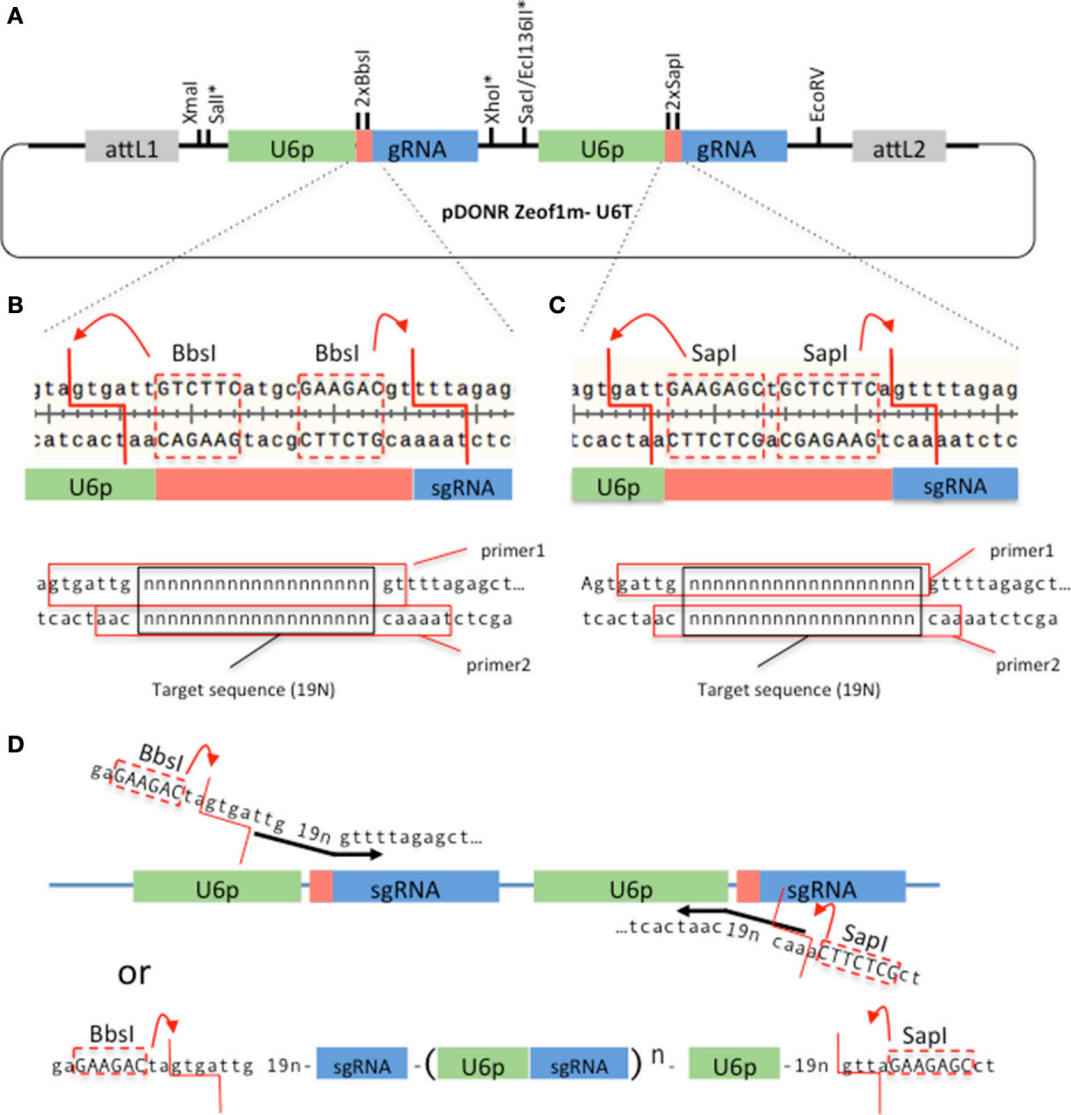
A Gateway-compatible vector for constructing U6promoter-sgRNA repeats. **(A)** The configuration of pDONRzeof1m-U6T vector. The first and second U6promoter-sgRNA repeat contains different type II restriction sites (BbsI or SapI) that allow seamless fusion of 19 bp gene-specific sequences to the vector. Asterisks indicate unique sites. The vector is compatible with GW-cloning, and can later be recombined with pMTN3164. **(B,C)**. Top panels: Regions around the gene specific sequences within the first and second U6-sgRNA repeats, respectively. Note that the type II sites are removed by the digestion. Bottom panels: Primers to introduce gene-specific sequences in the first and second sites, respectively. 19 “n”s represent the gene specific sequences. The red squares indicate the sequences of the top and bottom primers. **(D)** Alternative strategy to introduce the gene-specific sequences. In this case the forward primer carries a BbsI site, a 19 bp target sequence, and the beginning of the sgRNA sequence, whereas the reverse primer carries a SapI site, 19 bp target sequence, and the end of U6 promoter sequence. A PCR reaction is performed on the template of pDONRzeof1m-U6T vector. The resulting fragment is digested with BbsI and SapI, then cloned into the BbsI/SapI digested pDONRzeof1m-U6T vector. BbsI and SapI recognition sequences within the primers are indicated by upper cases.

We have developed a Gateway technology compatible vector, pDONRzeof1m-U6T (Figure 1A), that allows construction of a tandem U6promoter-sgRNA within a week. The gene-specific sequences can be synthesized as two pairs of complementary primers (Figures 1B,C), annealed and ligated with the pDONRzeof1m-U6T vector digested with BbsI and SapI (we have successfully performed a ligation involving four fragments; the vector, the fragment produced by the second BbsI site and the first SapI site, and the two annealed primers). Alternatively, forward and reverse primers that contain the target sequences and BbsI and SapI adaptors can be used to introduce gene-specific sequences. The resulting fragment can be digested with BbsI and SapI and ligated seamlessly into the pDONRzeof1m-U6T vector (Figure 1D). It is also possible to create more than two repeats by synthesizing multiple repeats, flanked by BbsI and SapI adaptors (Figure 1D). The resulting entry vector carrying the U6promoter-sgRNA repeats can be cloned into pMTN3164, a binary vector carrying the *CAS9* coding sequence tagged with the *N7* nuclear localization signal under the ubiquitin promoter of Arabidopsis (Supplemental Figure 2). PMTN3164 is a Gateway-compatible derivative of pCUT vector series, for which no off-target events were detected even when the whole genome of mutants generated using this vector was sequenced (Peterson et al., 2016). Non-detectable off-target effect could be attributed to a low level of CRISPR-Cas9 protein accumulation, which was found to be correlated with low off-target activities in other organisms (Hsu et al., 2013; Pattanayak et al., 2013; Peterson et al., 2016). Since pMTN3164 and pCUT vectors are identical except for the cloning site for sgRNA repeats, pDONRzeof1m-U6T vector offers a rapid gateway assembly into an expression system which offers a practically non-detectable off-target mutation rate.

### High resolution melting temperature analysis

High-resolution melting (HRM) curve analysis is routinely used in plant breeding to detect known polymorphisms (Simko, 2016). The sensitivity (capable of detecting single nucleotide polymorphism) is ideally suited for detecting small indels caused by the CRISPR/Cas9 system.

**Figure 2:**
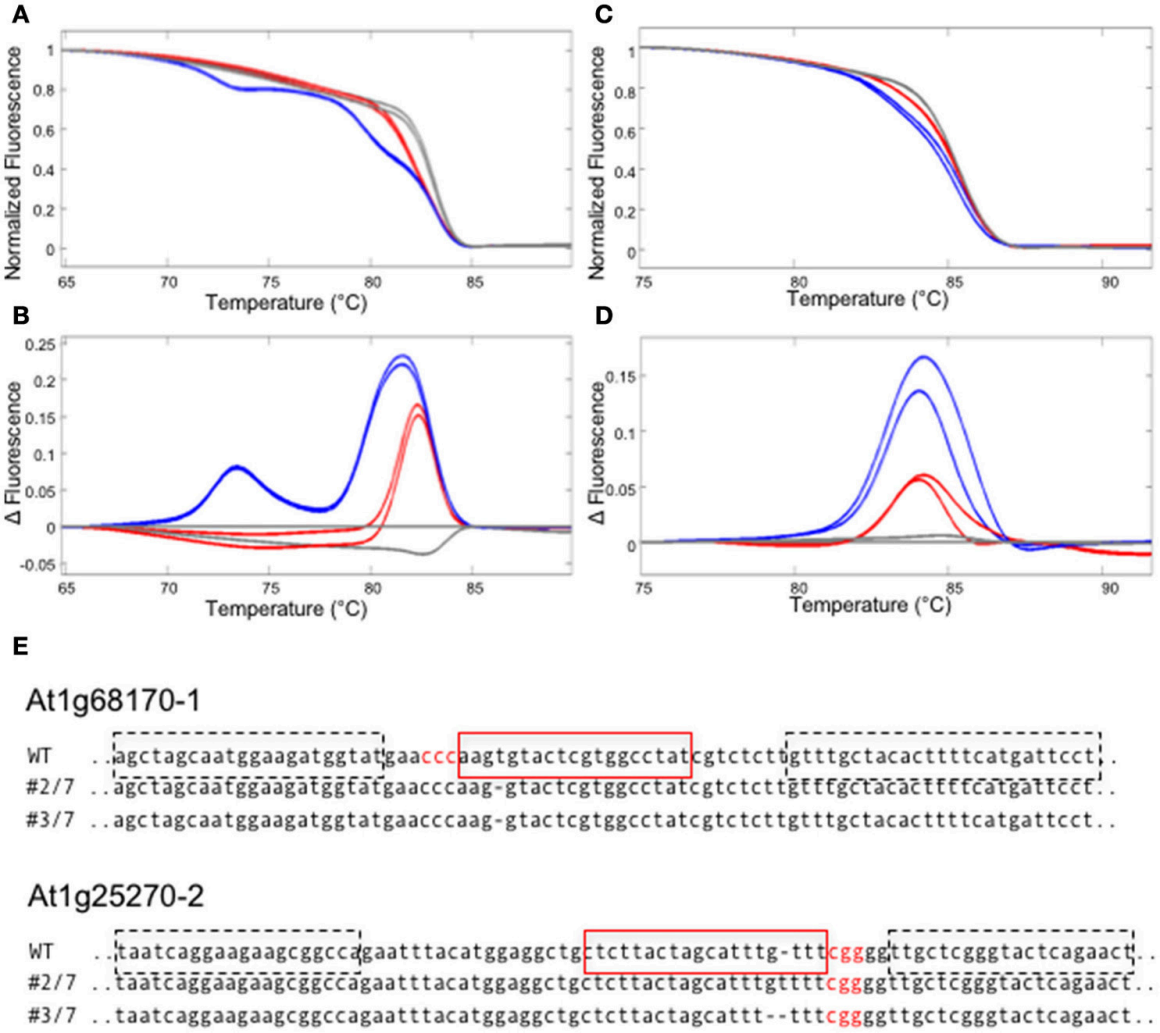
Detecting CRISPR/CAS9 mediated editing in T1 plants using HRM. **(A–D)** HRM analyses using the genomic DNA from T1 plants carrying sgRNAs against two Arabidopsis genes, At1g68170 and At1g25270. All experiments were performed in two technical replicates. **(A,B)** The melt curve **(A)** and the smoothed first derivatives of the melt curves, normalized to one of the WT samples **(B)** for the At1g68170-1 locus. Gray lines represent WT, whereas red and blue lines represent two independent transformed lines. **(C,D)** The melt curve **(C)** and the smoothed first derivatives of the melt curves, normalized to one of the WT samples **(D)** for the At1g25270-2 locus. **(E)** Homozygous mutations found in the progenies of plants shown in **(A–D)**. The target sequence is marked with a red square; PAM sequences are shown in red letters. Dotted squares indicate the positions of primers used for the analysis in **(A–D)**.

First, we have examined if gene editing activities can be detected in the T1 generation of Arabidopsis plants that express a nuclear-localized Cas9 protein and sgRNAs against two Arabidopsis genes (At1g68170 and At1g25270). For each target site, an amplicon that includes the target site was designed. The amplicon lengths ranging between 80 and 95 bp were chosen, because previous studies showed that amplicon sizes > 150 bp decrease sensitivity in HRM analysis (Gundry et al., 2003). GC contents of the amplicons ranged between 32 and 47% (Table S2). Previous studies report that high a GC content in the amplicon (>65%) could cause non-specific amplification, resulting in a multi-component melting curve that is hard to interpret (Laurie and George, 2009). Hence a care must be taken when the target gene is particularly GC rich. PCR reactions were performed on genomic DNA isolated from T1 Arabidopsis leaves, in the presence of LC Green Plus dye. HRM analyses revealed clear differences between theWT and T1 transgenic templates in three out of four target sites, enabling a quick detection of gene editing activity (Figures 2A–D and data not shown). We successfully isolated homozygous mutants for at least one target site per gene from the progenies of the T1 plants in which we detected gene editing activities (Figure 2E).

**Figure 3:**
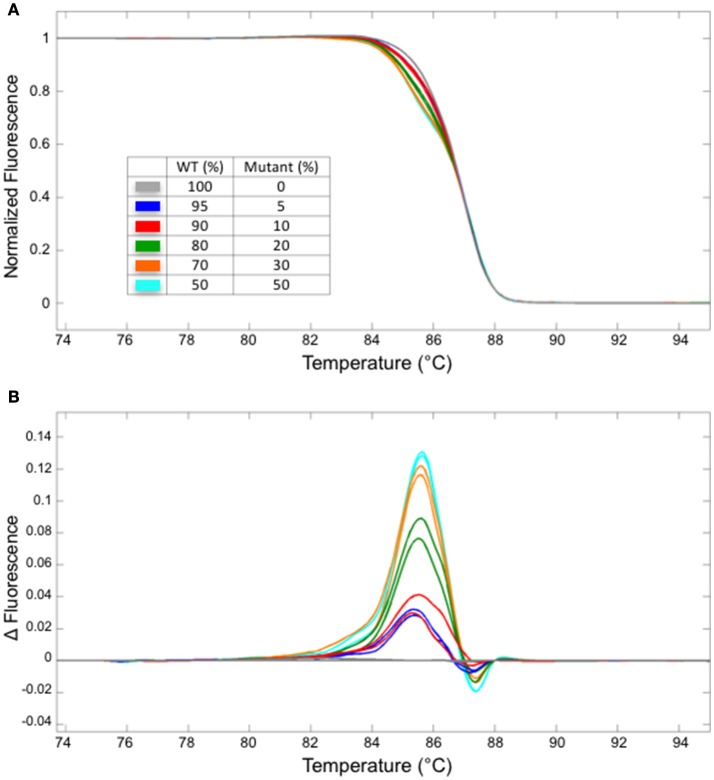
The detection limit for a fragment containing one bp insertion. Normalized melt curves **(A)** and the normalized, smoothed first derivatives **(B)** of the PCR fragments that were amplified from mixtures of mutant (line #2/7 shown in Figure 2) and WT DNA at varied ratios are shown. Different line colors represent various mutant/WT DNA ratios. All experiments were performed in two technical replicates. The line colors correspond to those shown in **(A)**.

Next, we have examined the detection limit of heteroduplex templates. For this purpose, genomic DNA from the WT and a homozygous mutant carrying one bp insertion in At1g25270 (line #2/7 in Figure 2E) were mixed at varied ratios, and used as a template for the PCR followed by HRM analysis. Melting curves from the sample with 5/95% mutant/WT template was clearly different from that of 100% WT, demonstrating that HRM analysis detects a small fraction of mutant DNA carrying one bp indel reliably (Figure 3). A similar result has been obtained for the mutation in At1g68170 (Supplemental Figure 1). The HRM analysis itself takes <10 min with no additional pipetting steps, offering a clear advantage over enzyme- or SDS-PAGE-based methods. Combined with genomic DNA extraction (~2 h) and PCR (~2 h), the whole procedure can be completed under 5 h.

**Figure 4:**
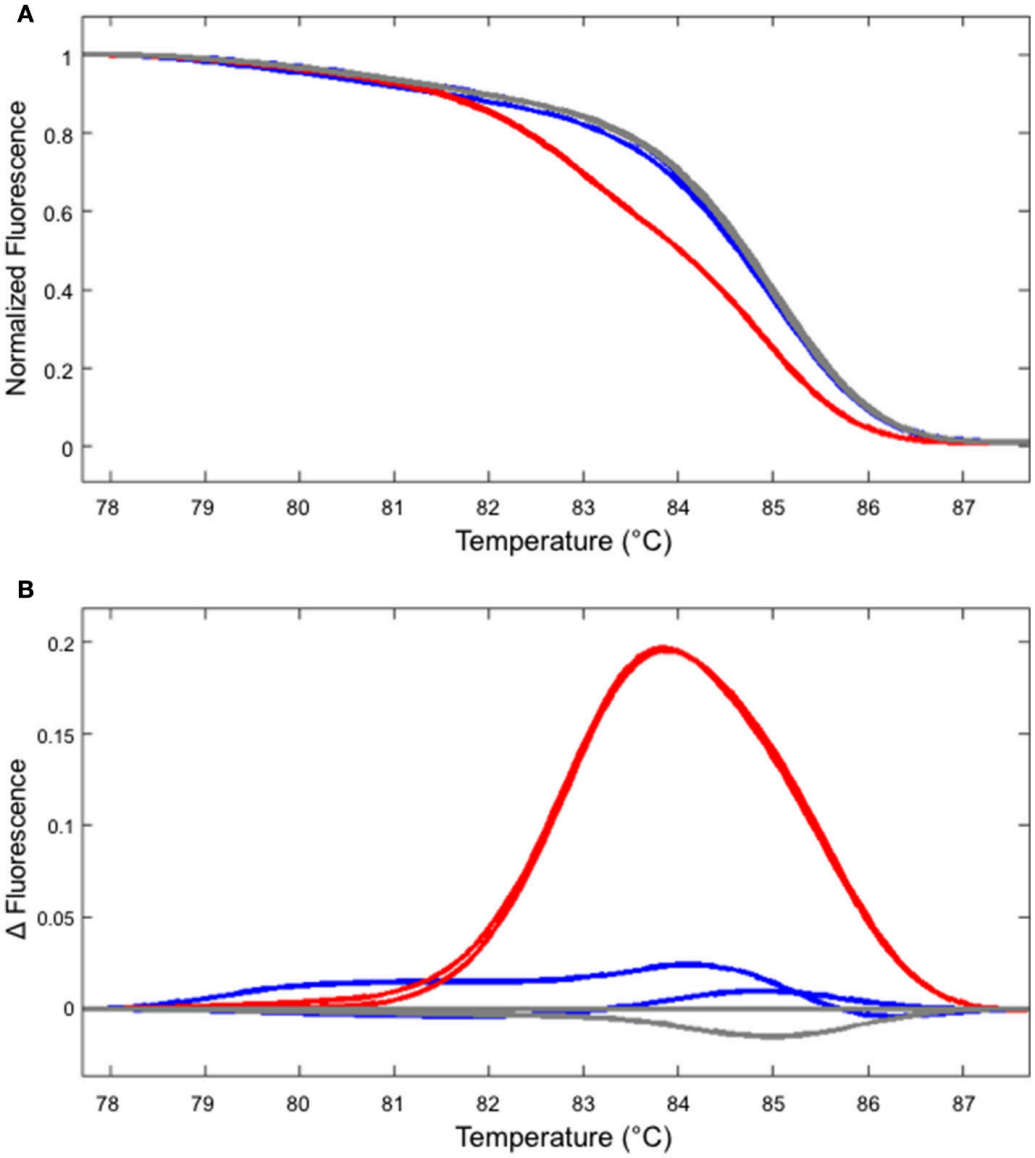
Identifying a homozygous mutant by performing two independent HRM analyses. **(A)** The melt curve produced by the WT (gray) and mutant DNA (line #2/7 shown in Figure 2, blue) are clearly distinguishable from the curve produced using the mixture of WT and mutant DNA (red). **(B)** The first derivatives of the melt curves, normalized to one of the WT samples. The colors of the lines correspond to those shown in **(A)**.

In our hands, HRM analysis did not distinguish WT and homozygous one bp insertion reliably (Figure 4 and data not shown). However, this shortcoming can be overcome by performing two PCR reactions, one with the genomic DNA from the potential homozygous mutant and another with WT DNA mixed at 1:1 ratio. In such a case, a clear shift in the melting temperature could be observed (Figure 4).

**Figure 5:**
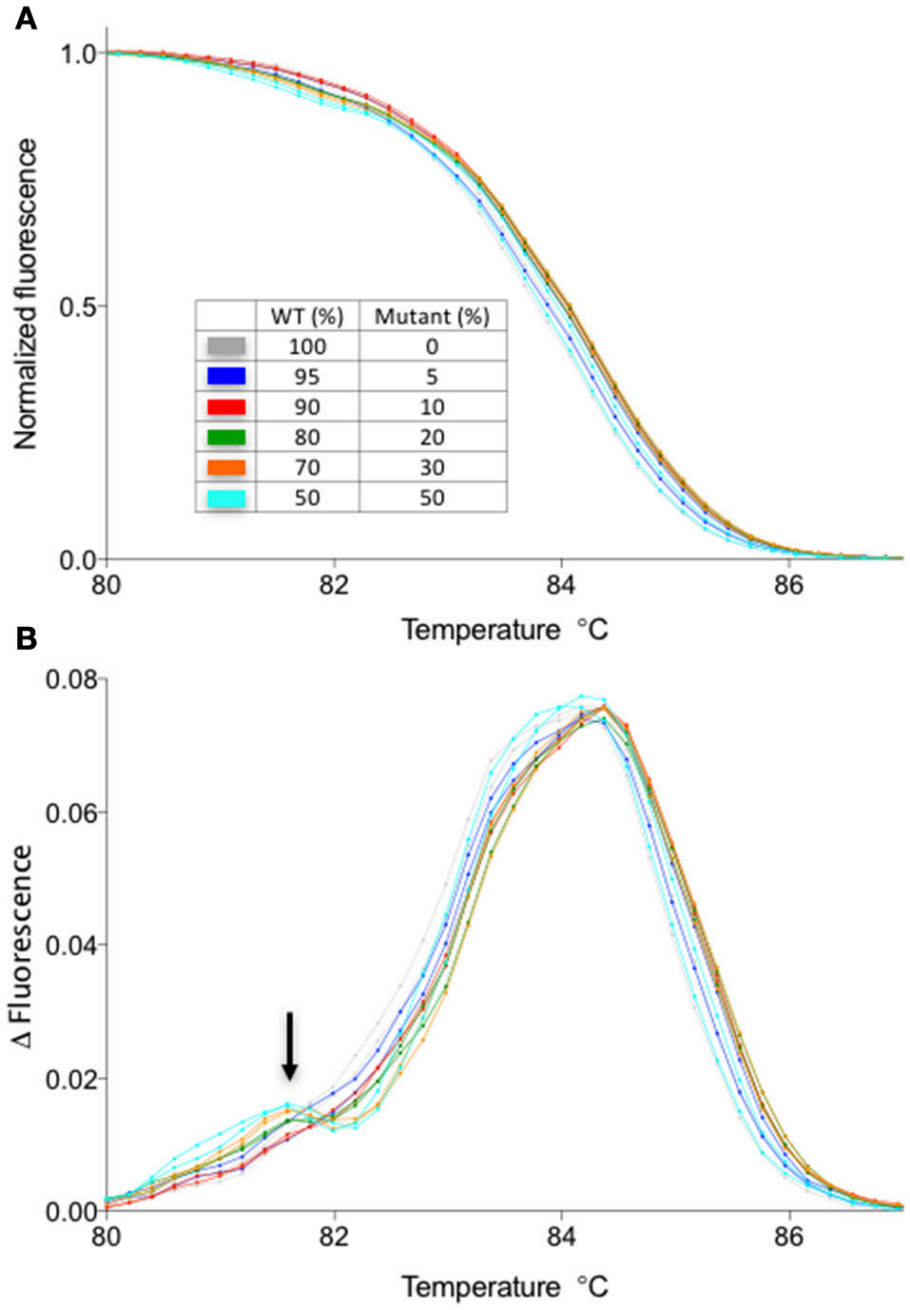
Melt curve analysis using LC Green plus dye in a qPCR machine without a high-resolution melt function. The samples are identical to those presented in Figure 3. **(A)** Normalized melt curves of the samples identical to those shown in Figure 3A. **(B)** Smoothed first derivative of the melt curve shown in the **(A)**. Note the appearance of a second peak in mixed samples (arrow). Normalization against the WT melt curve was not performed for this data set due to the increased noise in the data.

One disadvantage of HRM analysis is that it requires a dedicated hardware, whereas there might be a situation where an investigator does not have access to a machine capable of HRM analysis. Therefore, we have compared the results obtained by HRM analysis with a melting temperature analysis function of a regular RT-PCR machine. To do this, we have performed the melt curve analysis in a regular RT-PCR machine with the identical sample used in Figure 3. The results suggest that even though the sensitivity is decreased compared to HRM (detection limit is ~20% compared to 5% of HRM) and the experiment takes longer (>30 min), a shift in the melting temperature due to the heteroduplex formation could be observed by using a melt curve analysis function of a regular RT-PCR machine (Figure 5). Therefore, depending on the application, detection of gene editing activity might not require a dedicated hardware. For example, the T1 plant genotype is almost always mosaic, containing more than one type of deletion. In such a case, the shift in melting temperature curve is more prominent (see Figures 2A–D) than a situation in which the only type of mutation is a one bp indel. Therefore, the method described here could be useful in screening through a large number of T1 plants for individuals with gene editing activity. Combined with the flexibility of LC Green Plus dye that can be added to any PCR mixture of choice, the protocol shown here will offer a high-throughput detection of gene editing activities with a minimal change in a pre-existing PCR protocol. Also, while HRM analysis shown in this manuscript were performed in 96-well format, it is possible to scale up to a 384-well format as long as the detection system is compatible with a 384-well plate (e.g., either 384-well HRM system or qRT-PCR with 384-well detection).

## Author contributions

CD performed most of the HRM analysis and wrote the manuscript with SO. SL was involved in the analysis of T1 plants. ND and RS performed construction of vectors needed for the mutagenesis of two genes presented. ZN produced the gateway-compatible vector pMTN3164. SO designed and supervised the experiments, and wrote the manuscript.

### Conflict of interest statement

The authors declare that the research was conducted in the absence of any commercial or financial relationships that could be construed as a potential conflict of interest.
