# Supplementary material for: Gateway-Compatible CRISPR-Cas9 Vectors and a Rapid Detection by High-Resolution Melting Curve Analysis
Source: Front Plant Sci. 2017 Jul 5;8:1171. doi: 10.3389/fpls.2017.01171 (PMC5496963; doi:10.3389/fpls.2017.01171)
Supplement: Supplementary file 1 [file Table1.DOCX]

Table S1. Target sequence used for At1g68170 and At1g25270.

| Target | Strand | 19 bp Target sequences |
| --- | --- | --- |
| At1g68170-1 | bottom | ataggccacgagtacactt |
| At1g68170-2 | bottom | agctagacccgtaatggtt |
| At1g25270-1 | bottom | aaaaagagtaggaaacctt |
| At1g25270-2 | top | ctcttactagcatttgttt |
