# Supplementary material for: Gateway-Compatible CRISPR-Cas9 Vectors and a Rapid Detection by High-Resolution Melting Curve Analysis
Source: Front Plant Sci. 2017 Jul 5;8:1171. doi: 10.3389/fpls.2017.01171 (PMC5496963; doi:10.3389/fpls.2017.01171)
Supplement: Supplementary file 2 [file Table2.docx]

Table S2. Primers used in this study. Target sequences for At1g68170 and At1g27250 are indicated in red.

| Pair name | Strand | Sequence | Fragment size (bp) |
| --- | --- | --- | --- |
| At1g68170/27250-1 | Forward | aggaagactagtgattgataggccacgagtacacttgttttagagctagaaatagcaagt | - |
|  | Reverse | agagctcttcaaacaaggtttcctactctttttcaatcactacttcgactctagc |  |
| At1g68170/27250-2 | Forward | aggaagactagtgattgagctagacccgtaatggttgttttagagctagaaatagcaagt | - |
|  | Reverse | agagctcttcaaacaaacaaatgctagtaagagcaatcactacttcgactctagc |  |
| At1g68170-1 | Forward | agctagcaatggaagatggtat | 80 |
|  | Reverse | aggaatcatgaaaagtgtagcaaac |  |
| At1g68170-2 | Forward | cttctttaatgtggtgataattgaacagt | 95 |
|  | Reverse | agcagaggtgaatgttgctga |  |
| At1g25270-1 | Forward | tttcatgcttccccttgcc | 95 |
|  | Reverse | aggttatacattaaaaaatgcacactgat |  |
| At1g25270-2 | Forward | taatcaggaagaagcggcca | 82 |
|  | Reverse | agttctgagtacccgagcaa |  |
