# Supplementary material for: Gateway-Compatible CRISPR-Cas9 Vectors and a Rapid Detection by High-Resolution Melting Curve Analysis
Source: Front Plant Sci. 2017 Jul 5;8:1171. doi: 10.3389/fpls.2017.01171 (PMC5496963; doi:10.3389/fpls.2017.01171)
Supplement: Supplementary file 3 [file Image1.PDF]

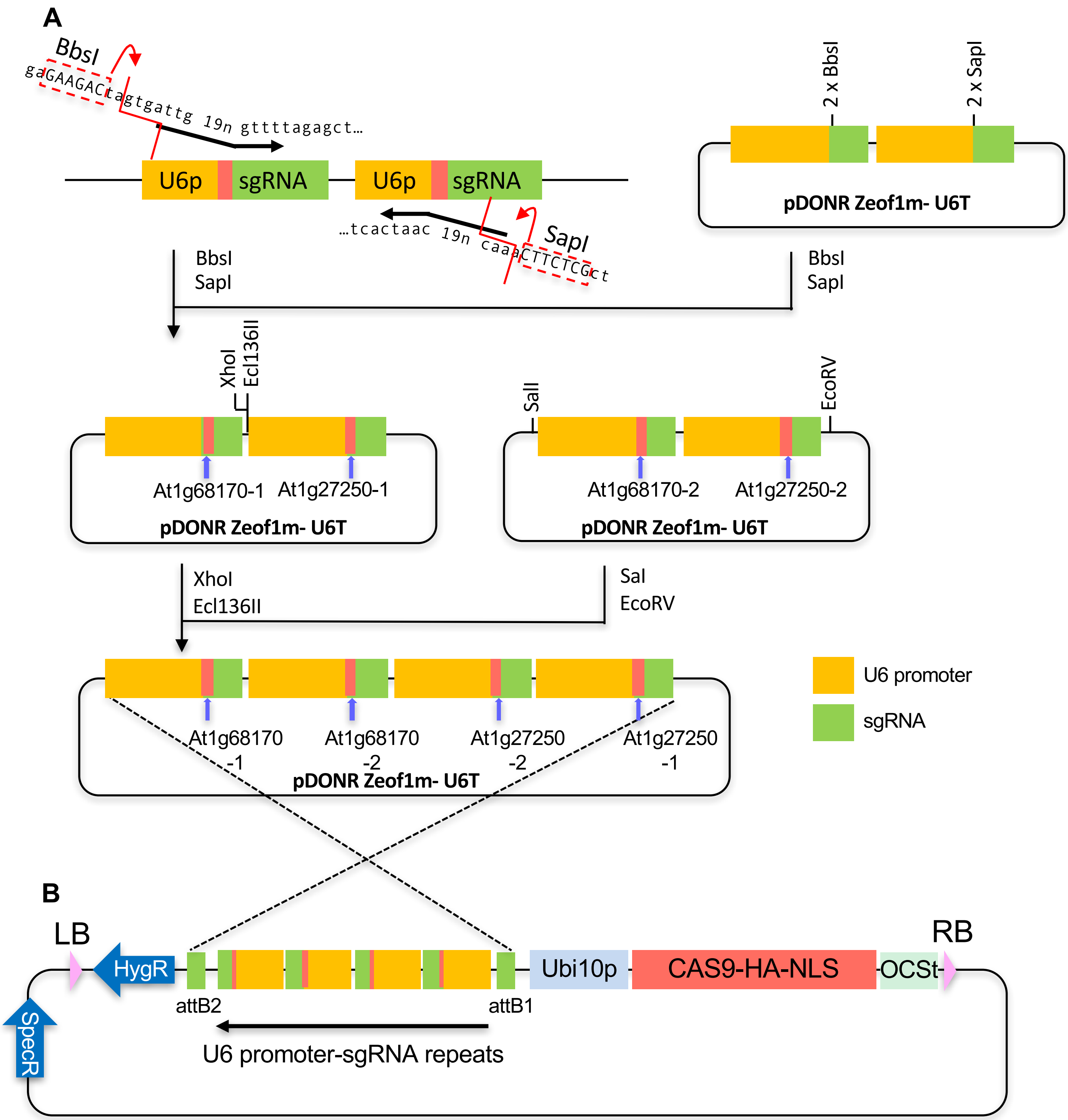

Supplemental Figure 1. A. Assembly of U6promoter-sgRNA repeats targeting At1g68170 and At1g27250. Two independent fragments containing one sgRNA targeting At1g68170, one U6 promoter and one target sequence (without the rest of the sgRNA) for At1g27250 were amplified by PCR and cloned into pDONR Zeof1m-U6T, which resulted in two complete U6-sgRNA units per construct. The two resulting constructs were then combined to produce a repeat of four U6promoter-sgRNA sequences. B. The plant binary vector used for introducing CRIPSR/CAS9 complexes targeting At1g68170 and At1g27250. LB: left border, RB: right border, attB1 and B2: Gateway recombination sites, Ubi10p: Ubiquitin 10 promoter, CAS9-HA-NLS: CAS9 protein tagged with HA tag and a nuclear localization signal, OCSt: octopine synthase terminator, HygR: hygromycin resistance cassette, SpecR: spectinomycin resistance cassette, sgRNA: single guide RNA. Gene specific target sequences are indicated by arrows.
