# Supplementary figures and images for: Gateway-Compatible CRISPR-Cas9 Vectors and a Rapid Detection by High-Resolution Melting Curve Analysis
Source: Front Plant Sci. 2017 Jul 5;8:1171. doi: 10.3389/fpls.2017.01171 (PMC5496963; doi:10.3389/fpls.2017.01171)

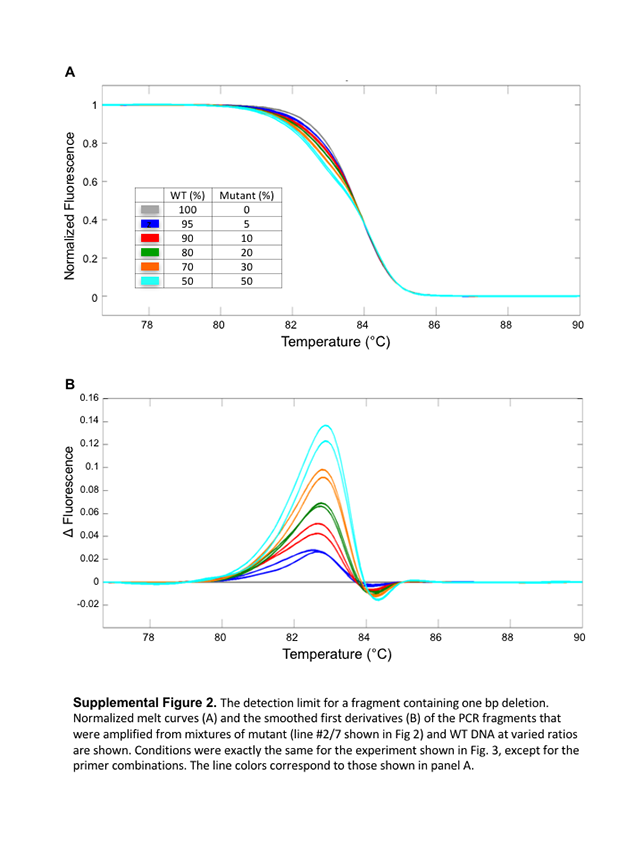

Supplement: Supplementary file 4 [file Image2.TIFF]
